# Structural Insights into the Stability of the Human STAT1–Nipah Virus V Protein Complex from Molecular Dynamics Simulations

**DOI:** 10.21315/mjms-02-2026-106

**Published:** 2026-06-30

**Authors:** Nur Syafiqah Mohamad Nasir, Zakuan Zainy Deris, Mohd Zulkifli Salleh

**Affiliations:** 1 Department of Medical Microbiology and Parasitology, School of Medical Sciences, Universiti Sains Malaysia, Health Campus, 16150 Kubang Kerian, Kelantan, Malaysia; 2 Hospital Pakar Universiti Sains Malaysia, Universiti Sains Malaysia, Health Campus, 16150 Kubang Kerian, Kelantan, Malaysia

**Keywords:** immune evasion, molecular docking, molecular dynamics, Nipah virus, STAT1, V protein

## Abstract

**Background:**

Nipah virus (NiV) phosphoprotein gene encodes multiple non-structural accessory proteins, including W, V, and C, which antagonise host innate antiviral defences by inhibiting interferon (IFN) signalling through suppression of interferon-stimulated genes (ISGs). The V protein has been reported to target host signal transducers and activators of transcription (STAT), particularly STAT1, by forming a high-molecular-weight STAT1/V assembly. However, the structural and dynamic behaviour of these assemblies remains poorly understood.

**Methods:**

In this study, we employed molecular docking and molecular dynamics (MD) simulations to compare the structural behaviour of apo-STAT1 with that of the STAT1/V complex. The NiV V peptide structure was first predicted using an ab initio Rosetta modelling approach before complex generation.

**Results:**

Computational modelling predicted a locally ordered core within the NiV V peptide (114–140). HADDOCK docking identified a dominant cluster (score −64.6 ± 11.2; Z-score −2.2) with strong electrostatic contributions and a 1278.7 Å^2^ buried surface area. MD analyses revealed that V binding enhances STAT1 structural stability and reduces conformational flexibility throughout the simulations.

**Conclusion:**

Collectively, these findings provide computational support for *in vitro* observations of stable STAT1/V assemblies and offer mechanistic insights into how NiV V binding interferes with IFN-β-induced STAT1 phosphorylation via the Src Homology 2 (SH2) domain.

## Introduction

Nipah virus (NiV) is a highly pathogenic zoonotic paramyxovirus first identified in 1998 in Sungai Nipah, Malaysia. To date, over 754 cases have been reported globally, with mortality rates exceeding 50%, primarily in South and Southeast Asia (1). Recent outbreaks in West Bengal, India, in January 2026 have prompted global health authorities to issue heightened precautions. The viral V protein, produced by co-transcriptional editing of the P gene, is a key antagonist of host interferon (IFN) signalling through disruption of the JAK–STAT pathway (2, 3). STAT1 and STAT2 are central transcription factors in type I and type III IFN signalling, undergoing phosphorylation-dependent dimerisation and nuclear translocation to induce interferon-stimulated genes (ISGs) (4, 5). The NiV V protein initially engages STAT1 via residues 114–140, targeting the STAT1 linker-Src homology 2 (SH2) domain, which is required for subsequent STAT2 recruitment (6, 7). Unphosphorylated STATs exist as latent dimers that undergo conformational rearrangements upon activation, a process that can be blocked by viral SH2-proximal binding (8). In the present study, we employed molecular docking and molecular dynamics (MD) simulations to investigate the conformational behaviour of the apo-STAT1 and STAT1/V complexes. Our results demonstrate that NiV V binding stabilises STAT1 and reduces its conformational flexibility, consistent with experimental evidence of the formation of stable, high-molecular-weight (HMW), non-productive NiV V-STAT complexes that impair STAT signalling (2, 7). This study aims to provide a structural and dynamic framework for understanding how NiV V-mediated stabilisation of the human STAT1 protein contributes to interferon antagonism, thereby offering mechanistic insights that may inform the rational design of antiviral strategies targeting the NiV–STAT protein interface.

## Methods

### Structure Retrieval and in Silico NiV V Peptide Modelling

The 3D structure of unphosphorylated human STAT1 protein (PDB ID: 1YVL; 3.00 Å resolution) was retrieved from the Research Collaboratory for Structural Bioinformatics Protein Data Bank (PDB). The NiV V peptide (residues 114–140) was modelled using the protein structure prediction server Robetta (https://robetta.bakerlab.org/, accessed on 20 December 2025) with the *ab initio* (RosettaAB) method. The model with the highest Global Distance Test and Template Modelling-score was selected for downstream analyses.

### Protein–Peptide Docking

Before docking, the STAT1 structure was manually inspected, and all water molecules and ligands were excluded. STAT1/V peptide docking was performed using the High Ambiguity Driven protein–protein DOCKing (HADDOCK v2.4) web server, available at https://rascar.science.uu.nl/haddock2.4/ (accessed on 21 December 2025), followed by high-resolution flexible-peptide refinement (FlexPepDock) in the Rosetta Online Server that Includes Everyone (ROSIE 2) available at https://r2.graylab.jhu.edu/apps/submit/flexpepdock (accessed on 22 December 2025). Docking simulations were conducted for a defined binding region—the SH2 domain region (residues 637–683) of STAT1 and the V peptide (residues 114–140)—using default parameters. The optimal docking model with the lowest *Z*-and HADDOCK scores, the largest HADDOCK v2.4 cluster, and the lowest Rosetta energy units (REU) in ROSIE 2 was selected for subsequent MD simulations. The interactions between STAT1 and the V peptide were visualised and analysed using PyMOL and PDBePISA (https://www.ebi.ac.uk/pdbe/pisa/; accessed on 22 December 2025).

### MD Simulation

MD simulations were performed using the GROningen MAchine for Chemical Simulations (GROMACS v2026.0) with the CHARMM 32 force field. The STAT1/V complex and apo STAT1 were solvated in a cubic TIP3P water model box, neutralised with 0.1 M NaCl, and energy-minimised using the steepest descent algorithm with a maximum of 50,000 steps and an energy tolerance of 1,000 kJ mol^−1^ nm^−1^. The V-rescale and Parinello-Rahman coupling methods were employed for the canonical ensemble (NVT) and isothermal-isobaric ensemble (NPT) equilibration, maintaining a constant temperature of 300 K. The equilibrated systems were prepared for the final production run of 100 ns. The structural coordinates were saved every 10 ps, and the resultant trajectories were analysed using GROMACS v2026.0, with plots generated in XMGRACE v5.1.25.

## Results

### NiV V Peptide Secondary Structure Prediction

The STAT-interacting region of the NiV V peptide (residues 114–140) was modelled using the Robetta protein structure prediction server, yielding a confidence score of 0.38. The model predicts a locally ordered central segment flanked by flexible termini. Per-residue error estimates were low across the central segment (approximately residues 8–20; <2 Å), suggesting a locally stable core, whereas higher predicted errors toward the N- and C-termini (up to ~3 Å) reflect increased terminal flexibility. Although this model is limited to the STAT-interacting peptide (residues 114–140), the predicted stable central core with flexible termini is consistent with features commonly observed in protein–protein interaction motifs, suggesting a potential mode of engagement with STAT proteins. PyMOL visualisation indicates that the NiV V peptide adopts a largely flexible, extended conformation with only short, isolated β-strand elements, consistent with a locally ordered core embedded within predominantly disordered regions (Figure 1).

### Protein–Peptide Docking Analysis

The NiV V peptide (residues 114–140) was docked to STAT1 using HADDOCK v2.4. For the data-driven method, all the interface residues of the V peptide were selected as active residues for docking. For the STAT1 protein interface, the SH2 domain region was selected as an active region (residues 637–683) (7). HADDOCK docking identified a dominant cluster (cluster 12) comprising five models with a favourable HADDOCK score (−64.6 ± 11.2) and a *Z*-score of −2.2, indicating a preferred binding mode. Structural convergence within the cluster was high, with a root mean square deviation (RMSD) of 0.7 Å. Complex stabilisation was driven mainly by electrostatic interactions (−289.4 ± 29.9 kcal/mol), with additional van der Waals contributions (−39.8 ± 3.4 kcal/mol). A substantial buried surface area (1278.7 ± 16.9 Å^2^) supported the formation of a stable protein–peptide interface. Relatively high restraint violation energies were observed (303.9 ± 48.1 kcal/mol). These are likely attributable to peptide flexibility and the use of limited or predicted interaction restraints—a common feature in protein–peptide docking analyses. To improve local geometry and hydrogen-bond interactions, the complex was further refined using FlexPepDock in the ROSIE 2 web server, allowing the peptide to adopt a conformation with more favourable interaction energies and reduced steric clashes and restraint violations. The model with the lowest reweighted score (−1,490.098 REU) and total score (−1,429.287 REU) was selected for subsequent MD simulations.

The analysis of the protein–protein interface using the PDBePISA server revealed a combination of hydrogen bonds and salt bridges stabilising the interaction between STAT1 and the NiV V peptide. Notably, glutamates at positions 9 and 20 of the NiV V peptide form hydrogen bonds with K652 and N662 of STAT1, with distances ranging from 1.61 Å to 3.00 Å, indicative of strong polar contacts contributing to complex stabilisation. The hydrogen bond involving Y26 of the NiV V peptide and N658 of STAT1 (1.90 Å) suggests the participation of side-chain hydroxyl groups in the recognition interface, potentially contributing to its specificity. In addition to hydrogen bonds, three salt bridges were also observed between glutamates of the NiV V peptide (positions 9 and 20) and K652 of STAT1, with distances between 2.63 Å and 3.66 Å (Figure 1b). These ionic interactions likely provide electrostatic complementarity, enhancing interface stability and rigidity. The convergence of multiple hydrogen bonds and salt bridges at the glutamate-lysine interface between the NiV V peptide and STAT1 highlights this region as a critical hotspot for binding, potentially essential for maintaining and stabilising the functional interaction. Interestingly, no disulphide or other covalent bonds were detected, suggesting that non-covalent forces predominantly drive the interaction. These hydrogen bond and salt bridge interactions align with previous observations in similar protein complexes, where polar and charged interactions play a dominant role in mediating specificity and stability without the need for covalent cross-links (7, 9).

### MD Analysis

MD simulations indicate that the NiV V peptide binding stabilises STAT1 at both global and local levels (Figure 2). Backbone RMSD analysis shows that the STAT1/V complex equilibrates rapidly and maintains a low RMSD over 100 ns, demonstrating enhanced global stability compared to the apo STAT1. Root mean square fluctuation (RMSF) analysis reveals that the local flexibility of STAT1 is markedly reduced upon V binding, particularly in the N-terminal domain (residues 1–136), where the STAT1 apo structure shows prominent peaks. The reduction of these RMSF peaks in the complex suggests that V binding restricts mobility in the N-terminal domain (NTD), likely stabilising the region critical for STAT1 dimerisation and interaction with other partners. Radius of gyration (Rg) analysis shows a transient increase during early equilibration, likely reflecting binding-induced conformational rearrangements, followed by stabilisation into a compact structure. Hydrogen bond analysis confirms the formation of persistent, dynamic interactions at the binding interface, with no evidence of dissociation. Collectively, these results indicate that binding of the NiV V protein constrains STAT1 dynamics at both global and local levels, as evidenced by reduced conformational flexibility in the NTD region. This restricted motion promotes the formation of a stable STAT1/V assembly. It is consistent with *in vitro* findings that NiV-derived peptides interfere with the host innate immune response by disrupting STAT protein activation (3, 7, 10, 11).

## Discussion

Accumulated structural and biochemical evidence indicates that the STAT proteins are highly dynamic and populate distinct conformational states in the cytoplasm (7, 10, 12). In their latent, unphosphorylated form, STAT1, either as a homodimer or in a heterodimer with other STATs, predominantly assembles into antiparallel dimers, stabilised by interactions between their NTDs (4, 5). Upon cytokine stimulation, tyrosine phosphorylation generates a phosphotyrosine site that is recognised by the SH2 domain, which promotes the formation of a parallel STAT1 dimer competent for nuclear translocation and transcriptional activation. Within this framework, the NiV V protein appears to exploit the intrinsic conformational plasticity of STAT complexes by targeting STAT1 before activation (7, 10). Our MD simulations demonstrate that binding of the V peptide to the STAT1 linker-SH2 region significantly stabilises STAT1, as reflected by reduced backbone deviations, decreased local flexibility, and suppressed global conformational fluctuations. This rigidification supports a model in which V binding restricts the structural rearrangements required for the antiparallel-to-parallel transition of STAT dimers, thereby preventing productive phosphotyrosine-SH2 engagement and downstream signalling (8, 13).

Although the present simulations focus on STAT1 alone, this interaction represents the initiating and essential step in NiV V-mediated antagonism of STAT signalling. Previous studies have demonstrated that STAT1 is required for V-dependent recruitment of STAT2, indicating that V does not independently target STAT2 but instead exploits pre-existing STAT1/STAT2 assemblies (10, 12). Stabilisation of STAT1 by V is therefore sufficient to compromise the functional integrity of the STAT1/STAT2 heterodimer, effectively trapping the complex in a non-signalling state. Consistent with experimental observations of the HMW STAT/V complexes, our results support a model in which V binding to STAT1 facilitates the formation of stable but transcriptionally inactive STAT assemblies.

In this study, comparative MD analyses reveal that STAT1 bound to V is more structurally stable than apo-STAT1, as evidenced by lower RMSD, reduced RMSF, a stabilised radius of gyration, and persistent hydrogen bonding. The transient increase in radius of gyration observed early in the simulation likely reflects binding-induced rearrangements rather than instability, followed by equilibration into a compact conformation. Together, these *in silico* findings support the broader paradigm of viral immune evasion, in which accessory proteins stabilise non-productive STAT conformations, sequester STATs in the cytoplasm, and inhibit their activation and nuclear translocation. The conformational stabilisation observed here provides a mechanistic explanation for the persistence of HMW STAT/V complexes reported experimentally and supports a mechanism by which NiV V suppresses interferon signalling by locking STAT complexes into an activation-incompetent state.

Collectively, sequestration of STAT1 by viral accessory proteins, particularly the NiV V protein, provides a mechanistic explanation for the profound suppression of innate antiviral responses, which correlates with rapid IFN antagonism and impaired STAT activation in infected cells (2, 10). Failure of STAT protein phosphorylation and nuclear translocation suppresses both type I and type III IFN signalling, thereby inhibiting downstream ISG induction. Clinically, this early and sustained suppression of IFN responses allows uncontrolled viral replication during the initial phase of infection, facilitating systemic dissemination to the central nervous system and respiratory tract, which are strongly associated with severe encephalitis, acute respiratory disease, and the high case fatality rates observed in NiV outbreaks (10, 14). Additionally, elevated and prolonged viral loads combined with immune antagonism increase the magnitude and duration of viral shedding in respiratory secretions, thereby enhancing the probability of transmission, particularly in close contact or healthcare settings.

Moreover, the multifunctional and structurally flexible NiV V protein complicates therapeutic intervention because it lacks intrinsic enzymatic activity and instead mediates immune evasion through dynamic protein–protein interactions with host signalling factors. The absence of a defined catalytic active site limits opportunities for classical small-molecule antiviral inhibition. At the same time, its reliance on adaptable host interactions poses challenges for rational antiviral and vaccine design (15).

## Conclusion

This study provides a molecular dynamics-based analysis that supports *in vitro* observations of the STAT1/V complex interaction and offers mechanistic insight into NiV immune evasion at the molecular level. Importantly, the MD simulations results from this study highlight the STAT1 linker–SH2 domain as a structurally vulnerable region exploited by the viral V protein, identifying a potentially targetable interface for therapeutic intervention. This work provides a computational framework for researchers to design inhibitors or peptides that disrupt STAT1–V interaction and restore interferon signalling for the treatment of NiV-infected patients.

## Figures and Tables

**Figure 1:**
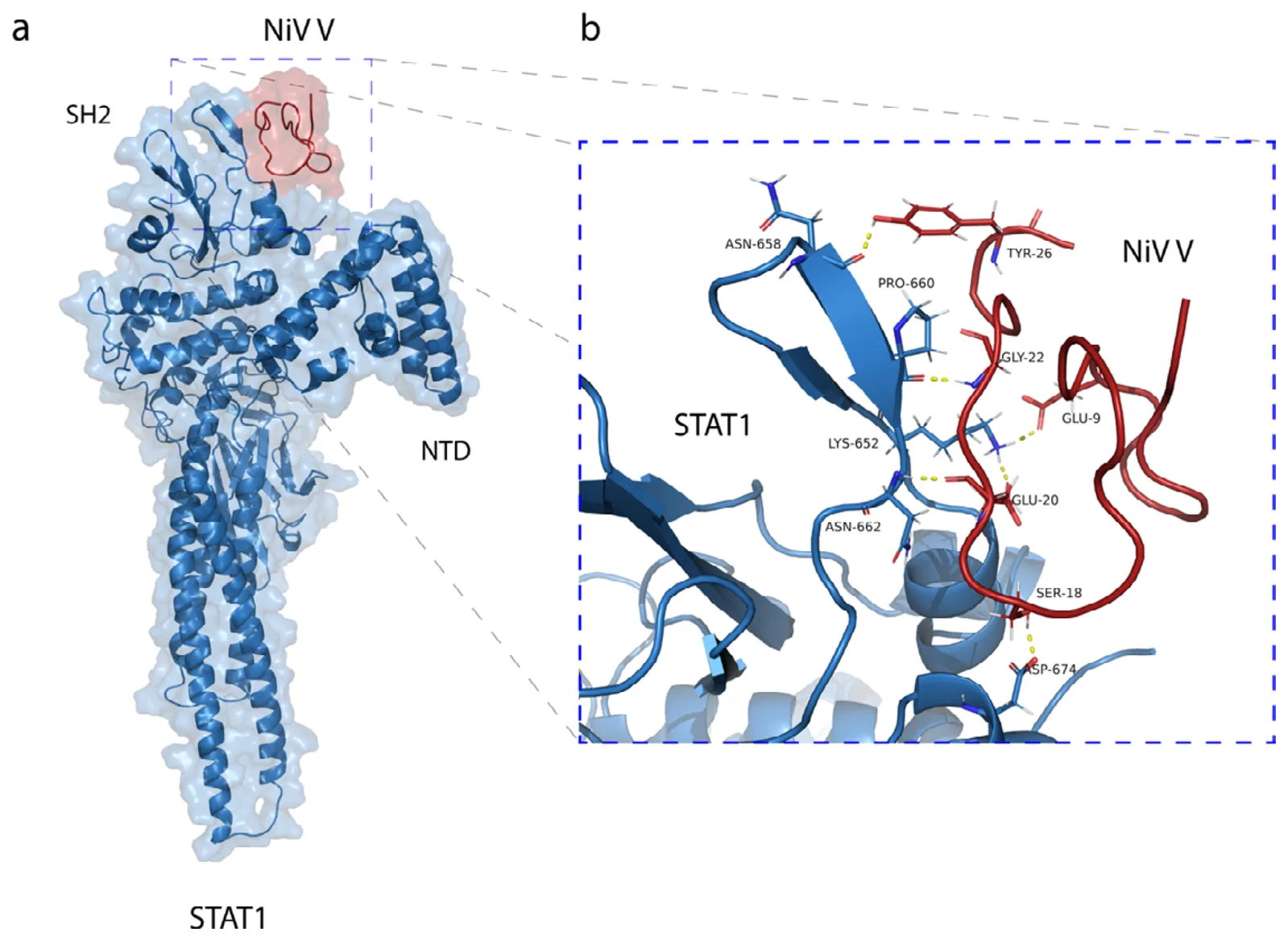
NiV V peptide bound to the STAT1 SH2 domain: a) Overall structure of STAT1 (blue) in complex with the NiV V peptide (red), showing the N-terminal domain (NTD) and the SH2 domain; b) Close-up view highlights hydrogen-bond interactions (yellow dashes lines) between the key residues of NiV V (SER18, GLU20, GLU9, GLY22, TYR26) and STAT1 (LYS652, ASN658, ASN658, PRO660, ASN662, ASP674), suggesting a mechanism for STAT1 antagonism by the NiV V protein.

**Figure 2:**
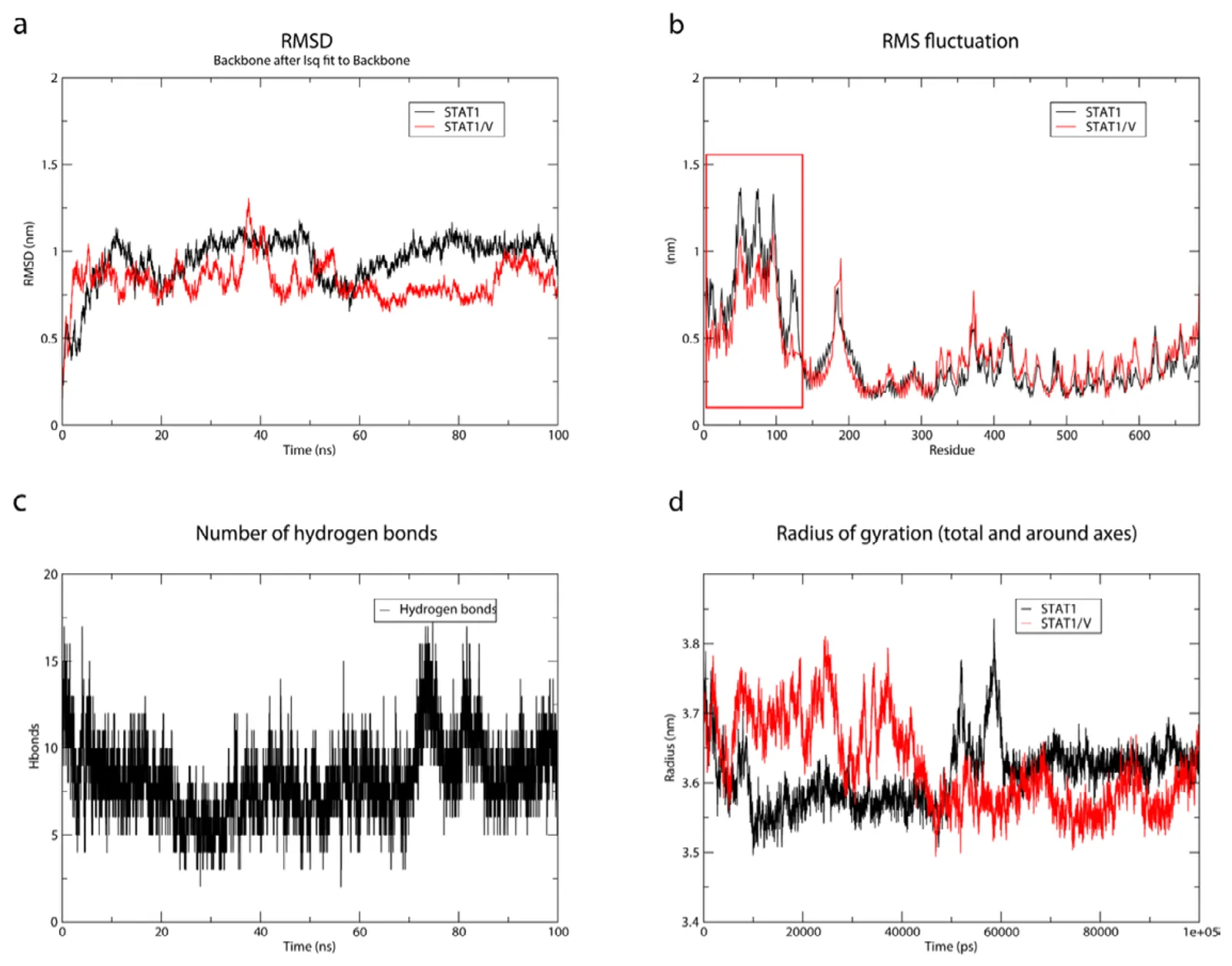
MD analysis of the apo STAT1and STAT1/V complex over 100 ns simulation: a) Root mean square deviation (RMSD) of STAT1 backbone atoms in the apo (free) and NiV V peptide-bound states; b) Root mean square fluctuation (RMSF) plot highlighting regions of decreased flexibility in the STAT1/V complex compared with the apo STAT1 (highlighted in red box), RMSF was calculated for the C*α* atoms of the STAT1 protein; c) Intermolecular H-bond analysis of the STAT1/V complex, indicating persistence peptide-protein interactions; d) Radius of gyration (Rg) plot showing comparable global compactness of STAT1 and the STAT1/V complex.
